# Online Access to Doctors' Notes: Patient Concerns About Privacy

**DOI:** 10.2196/jmir.2670

**Published:** 2013-09-26

**Authors:** Elisabeth Vodicka, Roanne Mejilla, Suzanne G Leveille, James D Ralston, Jonathan D Darer, Tom Delbanco, Jan Walker, Joann G Elmore

**Affiliations:** ^1^Harborview Medical CenterUniversity of Washington School of MedicineSeattle, WAUnited States; ^2^Beth Israel Deaconess Medical CenterHarvard Medical SchoolBoston, MAUnited States; ^3^College of Nursing and Health SciencesUniversity of MassachusettsBoston, MAUnited States; ^4^Group Health Research InstituteGroup Health CooperativeSeattle, WAUnited States; ^5^Geisinger Health SystemDanville, PAUnited States

**Keywords:** electronic medical records, patient access to records, patient portals, privacy, consumer health informatics, personal health records

## Abstract

**Background:**

Offering patients online access to medical records, including doctors’ visit notes, holds considerable potential to improve care. However, patients may worry about loss of privacy when accessing personal health information through Internet-based patient portals. The OpenNotes study provided patients at three US health care institutions with online access to their primary care doctors’ notes and then collected survey data about their experiences, including their concerns about privacy before and after participation in the intervention.

**Objective:**

To identify patients’ attitudes toward privacy when given electronic access to their medical records, including visit notes.

**Methods:**

The design used a nested cohort study of patients surveyed at baseline and after a 1-year period during which they were invited to read their visit notes through secure patient portals. Participants consisted of 3874 primary care patients from Beth Israel Deaconess Medical Center (Boston, MA), Geisinger Health System (Danville, PA), and Harborview Medical Center (Seattle, WA) who completed surveys before and after the OpenNotes intervention. The measures were patient-reported levels of concern regarding privacy associated with online access to visit notes.

**Results:**

32.91% of patients (1275/3874 respondents) reported concerns about privacy at baseline versus 36.63% (1419/3874 respondents) post-intervention. Baseline concerns were associated with non-white race/ethnicity and lower confidence in communicating with doctors, but were not associated with choosing to read notes or desire for continued online access post-intervention (nearly all patients with notes available chose to read them and wanted continued access). While the level of concern among most participants did not change during the intervention, 15.54% (602/3874 respondents, excluding participants who responded “don’t know”) reported more concern post-intervention, and 12.73% (493/3874 respondents, excluding participants who responded “don’t know”) reported less concern.

**Conclusions:**

When considering online access to visit notes, approximately one-third of patients had concerns about privacy at baseline and post-intervention. These perceptions did not deter participants from accessing their notes, suggesting that the benefits of online access to medical records may outweigh patients’ perceived risks to privacy.

## Introduction

Secure patient portals—tethered Web-based applications that enable patients to access their health information online—can give patients more control over their personal health information by improving their access to medical records [1-9]. A Markle Foundation survey of 1580 US adults in 2008 found that nearly half were interested in using an online patient portal and among those not interested, concern for privacy was the main deterrent to adoption [10]. While patients want easy access to their health information, including their doctors’ visit notes, concerns about the privacy of online medical data could limit the utility of patient portals [3,10,11]. Understanding patients’ views toward privacy is especially important in the case of visit notes, which often contain detailed personal information about patients’ medical, social, and family histories.

Despite calls for more discussion of patients’ privacy concerns in primary care settings [12-16], little research has addressed the concerns that arise when patients are given online access to their health information. Existing studies are primarily qualitative or opinion-based [8,17-19] or limited to a single health care institution [2,3,20,21]. Most have examined issues of privacy related to health information exchange or personal health information in general. None discuss privacy in the context of providing patients with electronic access to their visit notes.

This paper describes patient-reported concerns about privacy prior to and after participation in OpenNotes, a 1-year quasi-experimental study in which patients were offered online access to the outpatient clinic notes written by their primary care doctors (“visit notes”). Study procedures are fully described in prior publications and briefly summarized in the next section for context [22-25]. A priori, we generated several research questions to guide our analyses: What percentage of patients report concerns about privacy at baseline, and what are the characteristics of patients according to their level of concern? Did their attitudes change during participation in OpenNotes, and if so, in what direction? Were concerns about privacy at baseline associated with their use of visit notes, their likelihood of showing or discussing notes with others, or their desire for continued online access to notes after the intervention concluded?

## Methods

### Setting

We surveyed primary care doctors and their patients in three locations: Beth Israel Deaconess Medical Center (BIDMC), a teaching hospital of Harvard Medical School, and community practices affiliated with BIDMC in urban and suburban Boston, MA; Geisinger Health System (GHS), a rural integrated health services organization serving patients in central and northeastern Pennsylvania; and the adult medicine and HIV/AIDS clinics at Harborview Medical Center (HMC), a county hospital affiliated with the University of Washington that serves primarily safety-net populations in Seattle, WA. Each participating institution received approval for the study from its Institutional Review Board.

### Study Design

To be eligible for OpenNotes, patients at BIDMC and GHS had to be current users of their sites’ patient portals, through which they could message their doctors, schedule appointments, and view components of their medical records (such as medication lists and test results). OpenNotes patients at HMC gained first-time access to their hospital’s patient portal when they enrolled in the study. At all three sites, study participants gained first-time access to notes written by their doctors following the clinic visits that occurred during the intervention.

We surveyed patients both before and after the year-long OpenNotes study to gauge their attitudes toward and experiences with gaining online access to their visit notes. We used portal tracking data to confirm whether notes were generated for each participant during the intervention and whether participants chose to view notes that were available.

### Study Participants

For this report, we examined data from patients who completed the OpenNotes intervention, responded to both the baseline and 1-year post-intervention surveys, and completed the privacy questions on the surveys. We did not include patients who were excluded from participation (ie, denied online access to notes) by their doctors, who withdrew from the study, who moved or died during the intervention, or whose portal accounts became inactive during the study period. Information about the differences among patients by participation status and site has been published previously [22-25].

As described previously [23,25], 13,564 patients who completed the study had one or more notes made available during the year-long intervention period. Among those, 41.05% (5568/13,564) submitted a post-intervention survey. Further, 28.56% (3874/13,564) submitted both a baseline and a post-intervention survey with privacy questions completed; they constitute the study sample for this analysis.

### Patient Survey

Before developing the patient surveys, we conducted focus groups and individual interviews at the study sites to ensure that the surveys encompassed the major worries, expectations, and perceived benefits of online access to visit notes. Concern for the privacy of individual information was voiced by some patients during these focus groups and was particularly prevalent among patients at HMC [26]. The concerns about “privacy” that patients identified included login security, accessing their information in a public location (eg, library or hospital resource center), privacy breaches (eg, hackers or unauthorized hospital employees reading their medical information), and provision of their medical information to external organizations such as insurance companies and governmental agencies [26].

Based on focus group findings, we included an item addressing concerns about privacy in the baseline and post-intervention surveys, with responses on a 5-point Likert scale (baseline survey question: “If I could read my doctor’s notes, I would be concerned about my privacy: Agree, Somewhat Agree, Somewhat Disagree, Disagree, Don’t Know” and post-intervention survey question: “As a result of reading/having access to my doctor’s notes, I am concerned about my privacy: Agree, Somewhat Agree, Somewhat Disagree, Disagree, Don’t Know”).

In the baseline and post-intervention surveys, we also used the validated Ambulatory Care Experiences Survey (ACES) [27] and Perceived Efficacy in Patient-Physician Interactions (PEPPI) [28] instruments to assess participants’ level of trust in and interactions with their providers. The ACES instrument addresses patients’ self-reported quality of interaction and communication with their doctors, and PEPPI addresses levels of self-efficacy in communicating with doctors. Patients were also asked in the surveys to self-report demographic information including age, sex, race/ethnicity, education level, employment status, Internet use, and health status.

In the post-intervention survey, we asked patients a series of questions about whether or not they accessed their doctors’ notes (ie, “Did you look at any of your visit notes [on the secure patient portal]?”: Yes; No; I did not have any notes to look at because I did not see my doctor since notes were made available”), whether they would like OpenNotes to continue (ie, “I would like to continue to be able to see my doctor’s notes online: Yes, No”), and whether they shared their notes with others (ie, “Did you show or discuss your visit notes with other people? Yes, No, Don’t Know/Don’t Remember”).

As described in previous publications, we pre-tested the survey questions with patients for clarity and incorporated changes based on the patient feedback received. We conducted additional testing of online versions of the surveys prior to administering the surveys to participating patients [25]. See Multimedia Appendices 1 and 2 for the baseline and post-intervention survey instruments.

### Statistical Analysis

To assess patient characteristics associated with concerns about privacy, we performed a chi-square analysis on categorical variables, with age, race/ethnicity, employment, and self-reported health variables dichotomized for analytic purposes. To evaluate perceived confidence in doctor-patient communication and trust in doctors, we report quartile scores of the ACES and PEPPI summary measures. We also performed logistic regression models to determine whether age, sex, education, race/ethnicity, frequency of Internet use, and PEPPI scores were independently associated with the likelihood of having concerns about privacy at baseline. We performed McNemar’s test for paired data to determine whether patient attitudes toward privacy changed or persisted over the course of the intervention.

We analyzed data at both aggregated binary categories (collapsed categories into “Agree/Somewhat Agree” and “Disagree/Somewhat Disagree”) and disaggregated levels (“Agree”, “Somewhat Agree”, “Somewhat Disagree”, and “Disagree”). Unless otherwise stated, “concern for privacy” is reported as an aggregated percentage of both “Agree” and “Somewhat Agree” survey responses for improved clarity, and the findings were similar. For data that yielded significantly different results in the aggregate vs disaggregate, we report the findings separately. We used a confidence interval (CI) of 95% and defined statistical significance as a *P* value less than .05. All statistical analyses were conducted using SAS software, version 9.3.

## Results

### Privacy Concerns by Demographics

At baseline, about one-third of participants reported concerns about privacy related to online access to visit notes (Table 1). Compared to participants without such worries, they were more likely to be non-white, have fewer years of education (high school/GED or less), attend BIDMC, and report lower levels of trust and confidence in communication with their doctor. More modest associations were found according to age and sex (women worried more than men, under age 55 worried more than age 55 or older). We found no difference in levels of concern according to self-reported health status.

Following multivariable adjustment, differences according to gender, race/ethnicity, and confidence in communication remained significant (Table 2). Women were more likely to be concerned about privacy than men (adjusted OR 1.18, 95% CI 1.03-1.36). Non-white patients had greater concerns than white patients (adjusted OR 1.56, 95% CI 1.21-2.01). Patients who had less self-confidence about communicating with their doctors, based on their PEPPI scores, were more concerned about privacy than others who had more self-confidence about communication (adjusted OR 1.72, 95% CI 1.41-2.09 comparing lowest quartile to highest quartile of PEPPI score).

### Privacy Concerns and Use/Perception of OpenNotes

Baseline concerns about privacy were not associated with whether or not patients reported that they accessed their notes during the intervention or shared their notes with others (Table 3). Similarly, 99% of patients wanted continued access to their notes after the intervention concluded, regardless of concerns about privacy at baseline. At the end of the study, only 27 patients (1%) disagreed with the statement “having online access to my doctor’s notes is a good idea.” Among this very small subgroup, 8 patients (30%) were concerned about privacy.

### Privacy Concerns at Baseline and Post-Intervention

**Figure 1:**
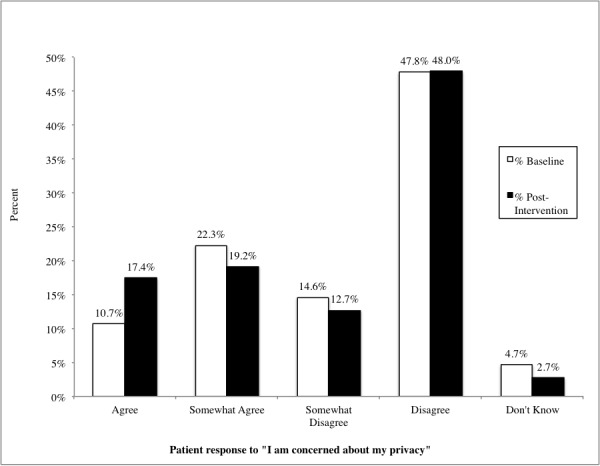
Patients' level of agreement with statements regarding concern about privacy on baseline and post-intervention surveys (N=3874).

We found a modest increase in reported concerns about privacy following the intervention, with 32.91% (1275/3874) of patients concerned at baseline, and 36.63% (1419/3874) concerned after the intervention (χ^2^_stat_=436.4; *P*<.001; Figure 1).

**Figure 2:**
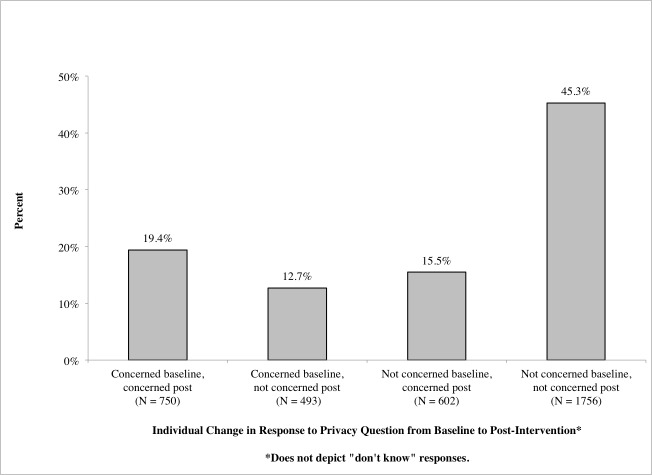
Change in individual patients' reported concern about privacy from baseline to post-intervention (N=3874).

For most patients, individual responses regarding privacy concern did not change over the course of the study period: 19% (750/3874) of patients reported concern at both baseline and post-intervention, and 45% (1757/3874) consistently reported none or little concern at baseline and post-intervention (Figure 2).

However, 28% (1095/3874) of patients reported changes in their level of concern from the beginning to the end of the study (see Multimedia Appendix 3 for individual patient responses at baseline versus their responses post-intervention). Patients who were concerned about privacy at baseline but not concerned post-intervention (12.73%, 493/3874) were slightly more likely to be younger or female compared to those whose level of concern remained unchanged (χ^2^_stat_=7.50; *P*=.006*;* χ^2^_stat_=8.63; *P*=.003; data not shown). In contrast, those whose attitudes shifted from being not concerned at baseline to being concerned post-intervention (15.54%, 602/3874) were slightly more likely to be older than those whose level of concern remained constant (χ^2^_stat_=16.66; *P*<.001; data not shown). Other attitudes and behaviors—for example, whether or not patients read their notes, wanted continued access to their notes, thought online access was a good idea, or shared their notes with others—were not significantly associated with changes in patients’ attitudes toward privacy (data not shown).

**Table 1:** Characteristics of patient respondents, stratified by baseline survey responses to statement: “If I could read my doctors’ notes, I would be concerned about my privacy” (N=3874).

Characteristics			Agree, %^a^	Somewhat agree, %^a^	Somewhat disagree, %^a^	Disagree, %^a^	Don’t know, %^a^	Total, n
Totals, n (%)			413 (10.66)	862 (22.25)	565 (14.58)	1853 (47.83)	181 (4.67)	3874 (100)
**Demographics**								
	**Age**							
		≥55 years old	11.89^c^	20.97	14.39	46.91	5.84	2036
		<55 years old	9.30	23.67	14.80	48.86	3.37	1838
	**Sex**							
		Female	11.12^b^	23.36	13.29	46.94	5.30	2303
		Male	9.99	20.62	16.49	49.14	3.76	1571
	**Race/Ethnicity**							
		White	9.94^c^	22.18	14.69	48.62	4.58	3540
		Non-White^e^	19.57	22.46	13.77	39.49	4.71	276
	**Education**							
		High school/GED or less	15.90^c^	19.20	12.89	45.85	6.16	698
		Some college	10.53	21.59	14.29	48.01	5.59	931
		College graduate	9.09	23.44	15.24	48.40	3.83	2244
	**Employment**							
		Employed	10.05^b^	22.99	15.13	48.01	3.81	2518
		Not employed	11.80	20.87	13.57	47.49	6.27	1356
	**Internet use**							
		Daily or almost daily	9.63^c^	22.34	15.02	48.91	4.11	3335
		>2 times per week	18.40	23.60	12.00	39.60	6.40	250
		Once per week	17.39	17.39	14.13	44.57	6.52	92
		Once every 2 weeks or less	16.79	19.71	11.68	40.88	10.95	137
		Not at all	14.71	20.59	5.88	50.00	8.82	34
	**Site**							
		Beth Israel Deaconess Medical Center	11.93^c^	25.44	14.71	43.36	4.55	2087
		Geisinger Health System	8.84	18.56	14.83	52.79	4.98	1686
		Harborview Medical Center	14.85	17.82	7.92	57.43	1.98	101
**Health & Health Care Experiences**								
	**Perceived confidence in communicating with physician (PEPPI)** ^d^							
		Q1 (Lowest confidence communicating with physician)	12.04^c^	26.54	16.71	39.56	5.16	814
		Q2	9.83	25.03	16.51	42.86	5.78	987
		Q3	10.70	22.56	16.37	46.38	3.99	953
		Q4 (Highest confidence communicating with physician)	10.34	16.28	9.80	59.62	3.96	1112
	**Perceived trust in physician score (ACES)**							
		<4.00 (Least trust in physician)	12.26^c^	28.42	16.52	38.54	4.26	563
		4.00-4.99	12.55	27.26	17.54	37.92	4.72	741
		5.00-5.99	9.65	21.33	15.00	48.56	5.47	1627
		6.00 (Greatest trust in physician)	10.03	15.55	10.47	60.75	3.20	907
	**Self-rated health status**							
		Good or excellent	10.74	22.55	14.46	47.71	4.55	3362
		Fair or poor	10.27	19.71	15.61	49.28	5.13	487

^a^Row percentages total 100%.

^b^Chi-square test for between group difference result *P*<.001.

^c^Chi-square test for between group difference result *P*<.01.

^d^Quartiles of PEPPI score (Perceived Efficacy in Patient-Physician Interactions); lower score indicates less self-confidence about communicating with their doctor [28].

^e^Non-White race/ethnicity categorized as aggregate of Black or African American; American Indian or Alaska Native; Asian; Native Hawaiian or Pacific Islander; Other.

**Table 2:** Associations of characteristics with baseline privacy concerns^a^ (N=3816).

Variable		Odds ratio	95% CI
**Age**			
	Under 55 years old	1.00	
	55 years old or older	1.04	(0.90-1.19)
**Sex**			
	Male	1.00	
	Female	1.18	(1.03-1.36)
**Race/Ethnicity**			
	White	1.00	
	Non-White	1.56	(1.21-2.01)
**Education**			
	College graduate	1.00	
	Some college	0.93	(0.79-1.10)
	High School/GED or less	1.07	(0.89-1.30)
**Frequency of Internet use**			
		
	Daily	1.00	
	Biweekly	1.53	(1.17-2.00)
	Once per week	1.12	(0.72-1.75)
	Every 2 weeks	1.18	(0.82-1.70)
	Not at all	1.09	(0.53-2.24)
**PEPPI** ^b^			
	Q4 (Highest confidence communicating with doctor)	1.00	
	Q3	1.40	(1.15-1.69)
	Q2	1.51	(1.25-1.83)
	Q1 (Lowest confidence communicating with doctor)	1.72	(1.41-2.09)

^a^Adjusted odds ratios from multivariable adjusted logistic regression models including all of the variables in the table; model estimates odds of patient responding “agree” or “somewhat agree” with statement: “If I could read my doctors’ notes, I would be concerned about my privacy” on the baseline survey.

^b^Quartiles of PEPPI score (Perceived Efficacy in Patient-Physician Interactions) [28].

**Table 3:** Post-intervention attitudes and behaviors regarding OpenNotes (N=3874).

		Total, n (%)	Agree/ Somewhat agree, n (%)	Disagree/ Somewhat disagree, n (%)	Don’t know, n (%)	*P* ^a^
Baseline survey privacy concerns		3874 (100)	1275 (32.91)	2418 (62.42)	181 (4.67)	
Post-survey question/ statement						
**OpenNotes is a good idea**						
	Agree/Somewhat agree	3828 (98.81)	1264 (99.14)	2387 (98.72)	177 (97.79)	.40
	Disagree/Somewhat disagree	27 (0.70)	8 (0.63)	17 (0.70)	2 (1.10)	
	Don’t know	19 (0.49)	3 (0.23)	14 (0.58)	2 (1.10)	
**Did you look at your visit notes?**						
	Yes	3832 (98.91)	1258 (98.67)	2394 (99.01)	180 (99.45)	.70
	No	41 (1.06)	17 (1.33)	23 (0.95)	1 (0.55)	
	Don’t know	1 (0.03)	0 (0.0)	1 (0.04)	0 (0.0)	
**Did you share or discuss your notes with others?**						
	Yes	796 (20.55)	240 (18.82)	520 (21.51)	36 (19.89)	.22
	No	3018 (77.90)	1017 (79.76)	1861 (76.96)	140 (77.35)	
	Don’t know	60 (1.55)	18 (1.41)	37 (1.53)	5 (2.76)	
**Do you want OpenNotes to continue?**						
	Yes	3834 (98.97)	1266 (99.29)	2389 (98.80)	179 (98.90)	.36
	No	40 (1.03)	9 (0.71)	29 (1.20)	2 (1.10)	

^a^ *P* values derived from chi-square test.

## Discussion

### Principal Findings

Overall, approximately one-third of patients at baseline reported concerns about privacy when considering gaining online access to their doctors’ visit notes. Nevertheless, this did not deter patients from accessing their notes and other medical information. Rather, nearly all patients wanted access to continue after the intervention despite the fact that nearly a third continued to worry about privacy. These findings are consistent with prior literature suggesting that patients want easy online access to their health information, despite concerns that might accompany such access [1,11,15,17,29].

The advancement of technologies such as patient portals carries the potential for a digital divide [30-36]. In our study sample that largely comprised experienced portal users, baseline concerns about privacy were more likely among individuals of non-white race/ethnicity, people reporting lower levels of self-confidence in communication, and those with less trust in their doctors. Lower education levels were also modestly associated with concerns about privacy. These associations are consistent with prior literature indicating that patients in these sociodemographic groups are, on the whole, less likely to enroll in patient portals and share personal health information [1,16,20,30-32,37,38]. Future research should consider whether worries regarding privacy might increase barriers to enrollment in patient portals among vulnerable populations, what factors associated with these sociodemographic groups contribute to privacy concerns, and how such concerns can be addressed.

Our study describes patients’ attitudes toward privacy before and after gaining online access to their doctors’ notes; however, it does not explore the reasons behind their concerns. The word “privacy” in itself carries different meanings for different people, and it may matter more to those who feel well than to individuals who are chronically or emergently ill [3,11]. But why did some patients become more concerned about privacy after a year’s experience with OpenNotes, while others became less concerned? Did their mode of access (eg, home computer, mobile device, shared computer in a library or other public space) influence levels of concern about privacy? As patient portals become more prevalent, doctors’ notes may become a common record component included in the information available to patients online. As such, how can doctors, administrators, and policy makers better address the future portal needs of patients, and how can they ensure that patients feel safe and secure logging on to read what the doctor has written?

### Limitations

While this research study gathered perspectives from 3874 patients at three diverse sites and included highly vulnerable patients, several limitations should be highlighted. First, participants at two sites (BIDMC and GHS) had been using patient portals before the study began and may be considered early and experienced adopters of such technology. Other literature demonstrates that individuals who use patient portals are typically less worried about privacy than nonusers [6,10], suggesting that the levels of concern reported by a substantial proportion of our respondents may not represent the general population. Nonetheless, while HMC patients received access to online health records for the first time through OpenNotes, they reported levels of concern on par with the average level of concern of registered portal users across study sites (approximately 33%; see Table 1).

In addition, the percentage of participants responding to both the pre- and post-intervention surveys was low, albeit consistent with other Internet-based surveys of patients [3]. And finally, while the topic of privacy was addressed in focus groups with patients before developing our survey [26] (a finding consistent with other focus group research on patient portals [39]) and the survey questions were vetted prior to administration, it is important to reiterate that the phrasing of the privacy question was used for the first time in the OpenNotes survey. Patients may not distinguish between the privacy risks of digitizing their health records (eg, hospital breaches of data security) and the risks of accessing those records online (eg, forgetting to log off a public computer, printing sensitive information). As a result, self-reported concerns about privacy may reflect diverse interpretations of potential associated risks.

### Conclusions

As patient portals and shared medical records proliferate, health professionals need to be aware of patients’ feelings about privacy. Our findings suggest that concerns about privacy among portal users do not deter them from accessing their visit notes and health information online. However, our findings also highlight the need to identify and address such concerns among specific demographic groups, particularly racial and ethnic minorities, patients with lower levels of education, and those with less trust in their doctors and lower confidence in their ability to communicate with them. If efforts to involve patients more actively in their care through Internet-based technologies are to move ahead, we need a far deeper understanding of the complex nature of “privacy” and how it interacts with the transparency that open visit notes represent.

## Multimedia Appendix 1

Baseline survey instrument.

## Multimedia Appendix 2

Post-intervention survey instrument.

## Multimedia Appendix 3

Individual patient matched baseline and post-intervention response to survey question: "I am concerned about my privacy" (N=3874).

## Abbreviations

ACES: Ambulatory Care Experiences Survey
BIDMC: Beth Israel Deaconess Medical Center
GED: General Educational Development test of high-school level academic skills
GHS: Geisinger Health System
HMC: Harborview Medical Center
OR: odds ratio
PEPPI: Perceived Efficacy in Patient-Physician Interactions score
