# Supplementary material for: Online Access to Doctors' Notes: Patient Concerns About Privacy
Source: J Med Internet Res. 2013 Sep 26;15(9):e208. doi: 10.2196/jmir.2670 (PMC3785972; doi:10.2196/jmir.2670)
Supplement: Supplementary file 1 [file jmir_v15i9e208_app1.pdf]

# OpenNotes Patient Survey

The OpenNotes project will begin at BIDMC in Summer 2010. During the 12-month project, patients of participating doctors will be able to read notes about their primary care office visits on PatientSite.

We ask that you complete the following survey before OpenNotes begins. We will ask you to complete another survey at the end of the project.

This survey will take about 10-20 minutes to complete. Your answers are confidential. Your doctor will not know how you answer the questions, and no one else in the practice or clinic you attend will know.  The research team will also not be able to identify you.

Please use the survey's "Back" or "Next" buttons (not your browser buttons) to move through the questionnaire.  When you are finished, click the “Submit Survey” button on the last page.

On behalf of the OpenNotes team, thank you!!

# Part 1: What you think about reading visit notes online

<b>After each office visit, a doctor writes a note in the patient's medical record that summarizes the visit. Usually patients do not see these visit notes. But in the future, patients may be able to view them on secure websites like PatientSite. <br>
<br>
The questions in this section ask what you think about this idea.</b>

1. In general, making visit notes available to patients on PatientSite is a good idea. (Required)
   - ◯ Disagree
   - ◯ Somewhat disagree
   - ◯ Somewhat agree
   - ◯ Agree
   - ◯ Don't know

2. If available, would you like to be able to look at your visit notes on PatientSite? (Required)
   - ◯ Yes
   - ◯ No
   - ◯ Don't know

3. Why would you like to be able to read your visit notes? (Check all that apply) (Required)
   - ☐ I am curious
   - ☐ I want to know about my health
   - ☐ I have a right to see what's in my medical record
   - ☐ I want to check the notes for accuracy
   - ☐ I want to see the diagnosis
   - ☐ Other (please specify) ________

4. Do you think you might show or discuss your visit notes with other people? (Required)
   - ◯ Yes
   - ◯ No
   - ◯ Don't know

5. With whom do you think you might share the note? (Check all that apply) (Required)
   - ☐ A family member or relative
   - ☐ A friend
   - ☐ Another doctor
   - ☐ A nurse or health professional
   - ☐ Someone else (specify relationship, no names please) ________

6. Why would you NOT like to be able to see your doctor's visit notes? (Check all that apply) (Required)

- ☐ I don't think it would be useful
- ☐ I do not use the internet very much
- ☐ I do not need to see what the doctor wrote about my visit
- ☐ I am afraid I will find out something bad about my health that I didn't know
- ☐ I do not want to think about my health any more than I have to
- ☐ No particular reason
- ☐ Other (please specify) ____________

7. It is important to me to be able to communicate with doctors and their offices by email. (Required)

- ◯ Disagree
- ◯ Somewhat disagree
- ◯ Somewhat agree
- ◯ Agree
- ◯ Don't know

# Part 2: How reading visit notes might affect you

<b>Please imagine what it might be like to read your doctor's notes about your office visits on PatientSite.</b>

If I could read my doctor's visit notes:

8. I would better understand my health and medical conditions. (Required)
- Disagree
- Somewhat disagree
- Somewhat agree
- Agree
- Don't know

9. I would better remember the plan for my care. (Required)
- Disagree
- Somewhat disagree
- Somewhat agree
- Agree
- Don't know

10. I would take better care of myself. (Required)
- Disagree
- Somewhat disagree
- Somewhat agree
- Agree
- Don't know

# Part 2 Continued: How reading visit notes might affect you

<b>Please imagine what it might be like to read your doctor's notes about your office visits on PatientSite.</b>

If I could read my doctor's visit notes:

11. I would be more likely to take my medications as prescribed. (Required)
- Disagree
- Somewhat disagree
- Somewhat agree
- Agree
- Don't know
- I don't take medications

12. I would feel more in control of my health care. (Required)
- Disagree
- Somewhat disagree
- Somewhat agree
- Agree
- Don't know

13. I would worry more. (Required)
- Disagree
- Somewhat disagree
- Somewhat agree
- Agree
- Don't know

# Part 2 Continued: How reading visit notes might affect you

<b>Please imagine what it might be like to read your doctor's notes about your office visits on PatientSite.</b>

If I could read my doctor's notes:

14. I would be concerned about my privacy. (Required)
- Disagree
- Somewhat disagree
- Somewhat agree
- Agree
- Don't know

15. The notes would be more confusing than helpful. (Required)
- Disagree
- Somewhat disagree
- Somewhat agree
- Agree
- Don't know

16. I would be better prepared for visits. (Required)
- Disagree
- Somewhat disagree
- Somewhat agree
- Agree
- Don't know

# Part 2 Continued: How reading visit notes might affect you

<b>Please imagine what it might be like to read your doctor's notes about your office visits on PatientSite.</b>

If I could read my doctor's notes:

17. It could make my doctor's job more difficult. (Required)

- ○ Disagree
- ○ Somewhat disagree
- ○ Somewhat agree
- ○ Agree
- ○ Don't know

18. If you have any other comments or thoughts about being able to read your visit notes online, please write them here:

# Part 3: The care you receive from your primary care doctor

<b>The following questions are about the care you receive from <u>your primary care doctor</u>. This is the doctor who takes care of you over time (not specialist doctors).</b>

19. How confident are you in your ability to know what questions to ask your doctor? (Required)
- Not at all Confident1
- 2
- 3
- 4
- 5
- 6
- 7
- 8
- 9
- Extremely Confident10

20. How confident are you in your ability to get your doctor to take your chief health concern seriously? (Required)
- Not at all Confident1
- 2
- 3
- 4
- 5
- 6
- 7
- 8
- 9
- Extremely Confident10

21. How confident are you in your ability to make the most of your visit with your doctor? (Required)
- Not at all Confident1
- 2
- 3
- 4
- 5
- 6
- 7
- 8
- 9
- Extremely Confident10

# Part 3 Continued: The care you receive from your primary care doctor

<b>The following questions are about the care you receive from <u>your primary care doctor</u>. This is the doctor who takes care of you over time (not specialist doctors).</b>

22. How confident are you in your ability to get your doctor to answer all of your questions? (Required)

- Not at all Confident1
- 2
- 3
- 4
- 5
- 6
- 7
- 8
- 9
- Extremely Confident10

23. How confident are you in your ability to get your doctor to do something about your chief health concern? (Required)

- Not at all Confident1
- 2
- 3
- 4
- 5
- 6
- 7
- 8
- 9
- Extremely Confident10

24. In the last 12 months, how often did your doctor explain things in a way that was easy to understand? (Required)

- Never
- Almost never
- Sometimes
- Usually
- Almost always
- Always
- I have not communicated with my doctor in the last 12 months

# Part 3 Continued: The care you receive from your primary care doctor

<b>The following questions are about the care you receive from <u>your primary care doctor</u>. This is the doctor who takes care of you over time (not specialist doctors).</b>

25. In the last 12 months, how often did your doctor listen carefully to you? (Required)

- ◯ Never
- ◯ Almost never
- ◯ Sometimes
- ◯ Usually
- ◯ Almost always
- ◯ Always

26. In the last 12 months, how often did your doctor or the staff give you clear instructions about what to do to take care of the health problems or symptoms that were bothering you? (Required)

- ◯ Never
- ◯ Almost never
- ◯ Sometimes
- ◯ Usually
- ◯ Almost always
- ◯ Always

27. In the last 12 months, how often did your doctor seem to know all the important information about your medical history? (Required)

- ◯ Never
- ◯ Almost never
- ◯ Sometimes
- ◯ Usually
- ◯ Almost always
- ◯ Always

# Part 3 Continued: The care you receive from your primary care doctor

<b>The following questions are about the care you receive from <u>your primary care doctor</u>. This is the doctor who takes care of you over time (not specialist doctors).</b>

28. In the last 12 months, how often did your doctor spend enough time with you? (Required)

- ○ Never
- ○ Almost never
- ○ Sometimes
- ○ Usually
- ○ Almost always
- ○ Always

29. In the last 12 months, how often did you feel you could tell your doctor anything, even the things that you might not tell anyone else? (Required)

- ○ Never
- ○ Almost never
- ○ Sometimes
- ○ Usually
- ○ Almost always
- ○ Always

# Part 3 Continued: The care you receive from your primary care doctor

<b>The following questions are about the care you receive from <u>your primary care doctor</u>. This is the doctor who takes care of you over time (not specialist doctors).</b>

30. Using any number from 0 to 10, where 0 is the worst doctor possible and 10 is the best doctor possible, what number would you use to rate your doctor? (Required)

- Worst possible doctor1
- 2
- 3
- 4
- 5
- 6
- 7
- 8
- 9
- Best possible doctor 10

31. Would you recommend your doctor to your family and friends? (Required)

- Definitely not
- Probably not
- Not sure
- Probably yes
- Definitely yes

# Part 4: About you

32. During the past 12 months, about how often did you use the Internet? (Required)
- ○ Not at all
- ○ About every two weeks or less
- ○ About once per week
- ○ 2 or more times per week but not every day
- ○ Daily or almost every day

33. Where do you use the Internet? (Check all that apply) (Required)
- ☐ Home
- ☐ Work
- ☐ Mobile telephone / Blackberry / iPhone
- ☐ Friend's home
- ☐ Public library
- ☐ School
- ☐ Other community setting (e.g. community center, resource center or senior center)
- ☐ Other (please describe) ______

34. How is your home computer connected to the Internet? (Required)
- ○ Broadband or Wireless (Cable, DSL, Fiber Optic, T-1, Satellite, 3G or cellular modem)
- ○ Dial-up telephone line
- ○ Other
- ○ Don't know or unsure

35. In general, how would you rate your overall health? (Required)
- ○ Excellent
- ○ Very Good
- ○ Good
- ○ Fair
- ○ Poor

36. What is the highest grade or level of school that you have completed? (Required)
- ○ 8th grade or less
- ○ Some high school, but did not graduate
- ○ High school graduate or GED
- ○ Some college or 2-year degree
- ○ 4-year college graduate
- ○ Some post-baccalaureate graduate school
- ○ Masters or Doctoral degree

37. Are you of Spanish/Hispanic/Latino ethnicity?
- ○ Yes
- ○ No

38. What do you consider to be your racial background? (Check all that apply)
- ☐ White
- ☐ Black or African American
- ☐ American Indian or Alaskan Native
- ☐ Asian
- ☐ Native Hawaiian or Pacific Islander
- ☐ Other

39. Which of the following best describes your current employment status? (Check all that apply)
- ☐ Employed for wages
- ☐ Self-employed
- ☐ Homemaker
- ☐ Unemployed
- ☐ Retired
- ☐ Unable to work
- ☐ Prefer not to answer

40. If there is anything else that you would like us to know about you, or other comments about OpenNotes you would like to make, please write them here:
