# Supplementary material for: Online Access to Doctors' Notes: Patient Concerns About Privacy
Source: J Med Internet Res. 2013 Sep 26;15(9):e208. doi: 10.2196/jmir.2670 (PMC3785972; doi:10.2196/jmir.2670)
Supplement: Supplementary file 2 [file jmir_v15i9e208_app2.pdf]

# OpenNotes Survey

## Welcome to the OpenNotes Survey!

Please use the survey's "Back" or "Next" buttons (not your browser buttons) to move through the questionnaire. When you are finished, click the “Submit Survey” button on the last page.

You may leave the survey and return to finish at a later time by clicking the "Save and continue survey later" link located in the middle at the top of your computer screen. You will need to supply an email address to save your progress. A link will be emailed to you that will allow you to return to your survey where you left off. When you are ready to continue your survey, simply click on the link in the email.

Please click the “Next” button to begin the survey.

---

## What you think about reading visit notes online

In general, making visit notes available to patients on PatientSite is a good idea. *

- ○ Disagree
- ○ Somewhat disagree
- ○ Somewhat agree
- ○ Agree
- ○ Don't know

---

Did you look at any of your visit notes on PatientSite? *

- ○ Yes
- ○ No
- ○ I did not have any notes to look at because I did not see my doctor since notes were made available (June 2010).

---

## What you think about reading visit notes online

What was the reason you did not look at your doctor's visit notes? *(Check all that apply)* *

- [ ] I didn't think it would be useful
- [ ] I forgot my notes were available
- [ ] I tried, but I could not find my notes on PatientSite
- [ ] I was too busy
- [ ] I thought reading the notes would make me nervous or anxious
- [ ] Another reason
- [ ] No particular reason

---

Please tell us more about why you didn't read your doctor's notes.

---

## What you think about reading visit notes online

Why did you read your visit notes? *(Check all that apply)* *

- [ ] I was curious
- [ ] I wanted to remember what happened in the visit
- [ ] I wanted to know about my health
- [ ] I have a right to see what's in my medical record
- [ ] I wanted to check the notes to see if they were right
- [ ] I wanted to be sure I understood what the doctor said
- [ ] I wanted to know what my doctor was thinking
- [ ] No particular reason
- [ ] Other reason (please explain)

---

Did you show or discuss your visit notes with other people? *

- ○ Yes

- ○ No
- ○ Don't know/don't remember

## What you think about reading visit notes online

With whom did you share or discuss the note? *(Check all that apply)* *

- ☐ Another doctor
- ☐ A family member/relative/friend who helps take care of me
- ☐ Another family member or relative
- ☐ Another friend
- ☐ A nurse or health professional
- ☐ Someone else (specify relationship, no names please)

## What you think about reading visit notes online

Did it help you to share your notes with family members or friends who are helping with your health care needs? *

- ○ Yes
- ○ No

## What you think about reading visit notes online

I would like to continue to be able to see my doctor's notes online. *

- ○ Yes
- ○ No

## What it was like to read your visit notes

**Please think about what it was like to read your doctor's notes about your office visits on PatientSite.**

How often were the doctor's notes put on PatientSite as soon as you wanted them? *

- Never
- Sometimes
- Usually
- Always
- Don't know

How often did the notes accurately describe the visit? *

- Never
- Sometimes
- Usually
- Always
- Don't know

# What it was like to read your visit notes

How easy was it to understand your doctor's notes? *

- Very difficult
- Somewhat difficult
- Somewhat easy
- Very easy
- Don't know

Did you ever mention to your doctor that you had read his/her note online? *

- Yes
- No
- Don't know/don't remember

Did you ever contact your doctor's office about something in your notes? *

- ○ Yes
- ○ No, I did not feel any need to
- ○ I considered contacting my doctor's office but decided not to
- ○ Don't know/don't remember

## What it was like to read your visit notes

Why did you decide not to contact your doctor's office? *(Check all that apply)* *

- ☐ I didn't want to waste my doctor's time
- ☐ I didn't think it was important
- ☐ I didn't want my doctor to be angry with me
- ☐ I worried my doctor might not take as good care of me
- ☐ I worried my doctor might get back at me
- ☐ It was too much of a bother for me
- ☐ Another reason (please explain)

## What it was like to read your visit notes

Why did you contact your doctor's office about something in your notes? *(Check all that apply)* *

- ☐ I wanted an explanation of something in my notes
- ☐ I wanted something removed from my permanent record
- ☐ I wanted to report something I thought was an error in my notes
- ☐ I wanted to discuss something I disagreed with
- ☐ Another reason (please explain)

Were you satisfied with the doctor's or office's response to your request? *

- ○ Yes

Somewhat

No

## What it was like to read your visit notes

Please briefly tell us about how you used your notes. We would appreciate any examples you have to help us understand.

## What it was like to read your visit notes

### As a result of reading/having access to my doctor's notes...

I understand my health and medical conditions better. *

Disagree

Somewhat disagree

Somewhat agree

Agree

Don't know

I remember the plan for my care better. *

Disagree

Somewhat disagree

Somewhat agree

Agree

Don't know

I take better care of myself. *

- Disagree
- Somewhat disagree
- Somewhat agree
- Agree
- Don't know

## What it was like to read your visit notes

I do better with taking my medications as prescribed. *

- Disagree
- Somewhat disagree
- Somewhat agree
- Agree
- Don't know
- I don't take medications

I feel more in control of my health care. *

- Disagree
- Somewhat disagree
- Somewhat agree
- Agree
- Don't know

I worry more. *

- Disagree
- Somewhat disagree
- Somewhat agree
- Agree

- Don't know

## What it was like to read your visit notes

I am concerned about my privacy. *

- Disagree
- Somewhat disagree
- Somewhat agree
- Agree
- Don't know

I felt offended.

- Disagree
- Somewhat disagree
- Somewhat agree
- Agree
- Don't know

## What it was like to read your visit notes

The notes are more confusing than helpful. *

- Disagree
- Somewhat disagree
- Somewhat agree
- Agree
- Don't know

I am better prepared for visits. *

- Disagree

- Somewhat disagree
- Somewhat agree
- Agree
- Don't know

## What it was like to read your visit notes

Did reading the doctor's notes change the way you feel about your doctor?
*

- I feel much worse
- I feel somewhat worse
- I don't feel better or worse
- I feel somewhat better
- I feel much better

Did something happen (good or bad) as a result of reading your notes? Please describe your experience here.

## Thinking about your visit notes

Since this is a research project, the future of continued patient access to doctor’s notes is uncertain. How would you feel personally if open notes were turned off? *

- Very pleased- I would like to stop being able to read them online
- Somewhat pleased
- Would not care
- Somewhat disappointed
- Very disappointed- I do not want my online access to the notes turned off

In the past, have you ever requested a copy of your medical records from your doctor's office? *

- Yes
- No

## Thinking about your visit notes

If Yes, please tell us about the experience of getting your records (was it easy, difficult, etc.)

## The care you receive from your primary care doctor

**The following questions are about the care you receive from your primary care doctor. This is the doctor who takes care of you over time (not specialist doctors).**

How confident are you in your ability to know what questions to ask your doctor? *

| Not at all Confident | | | | | | | | | Extremely Confident |
|---|---|---|---|---|---|---|---|---|---|
| 1 | 2 | 3 | 4 | 5 | 6 | 7 | 8 | 9 | 10 |

How confident are you in your ability to get your doctor to take your chief health concern seriously? *

| Not at all Confident | | | | | | | | | Extremely Confident |
|---|---|---|---|---|---|---|---|---|---|
| 1 | 2 | 3 | 4 | 5 | 6 | 7 | 8 | 9 | 10 |

How confident are you in your ability to make the most of your visit with your doctor? *

| Not at all Confident | | | | | | | | | Extremely Confident |
|---|---|---|---|---|---|---|---|---|---|
| 1 | 2 | 3 | 4 | 5 | 6 | 7 | 8 | 9 | 10 |

## The care you receive from your primary care doctor

How confident are you in your ability to get your doctor to answer all of your questions? *

| Not at all Confident | | | | | | | | | Extremely Confident |
|---|---|---|---|---|---|---|---|---|---|
| 1 | 2 | 3 | 4 | 5 | 6 | 7 | 8 | 9 | 10 |
| ○ | ○ | ○ | ○ | ○ | ○ | ○ | ○ | ○ | ○ |

How confident are you in your ability to get your doctor to do something about your chief health concern? *

| Not at all Confident | | | | | | | | | Extremely Confident |
|---|---|---|---|---|---|---|---|---|---|
| 1 | 2 | 3 | 4 | 5 | 6 | 7 | 8 | 9 | 10 |
| ○ | ○ | ○ | ○ | ○ | ○ | ○ | ○ | ○ | ○ |

## The care you receive from your primary care doctor

In the last 12 months, how often did your doctor explain things in a way that was easy to understand? *

- ○ Never
- ○ Almost never
- ○ Sometimes
- ○ Usually
- ○ Almost always
- ○ Always
- ○ I have not communicated with my doctor in the last 12 months

## The care you receive from your primary care doctor

In the last 12 months, how often did your doctor listen carefully to you? *

- ○ Never
- ○ Almost never

- Sometimes
- Usually
- Almost always
- Always

---

In the last 12 months, how often did your doctor or the staff give you clear instructions about what to do to take care of the health problems or symptoms that were bothering you? *

- Never
- Almost never
- Sometimes
- Usually
- Almost always
- Always

---

## The care you receive from your primary care doctor

In the last 12 months, how often did your doctor seem to know all the important information about your medical history? *

- Never
- Almost never
- Sometimes
- Usually
- Almost always
- Always

---

In the last 12 months, how often did your doctor spend enough time with you? *

- Never
- Almost never
- Sometimes

- Usually
- Almost always
- Always

In the last 12 months, how often did you feel you could tell your doctor anything, even the things that you might not tell anyone else? *

- Never
- Almost never
- Sometimes
- Usually
- Almost always
- Always

## The care you receive from your primary care doctor

Using any number from 1 to 10, where 1 is the worst doctor possible and 10 is the best doctor possible, what number would you use to rate your doctor? *

| **Worst possible doctor** | | | | | | | | | **Best possible doctor** |
|---|---|---|---|---|---|---|---|---|---|
| 1 | 2 | 3 | 4 | 5 | 6 | 7 | 8 | 9 | 10 |
| ○ | ○ | ○ | ○ | ○ | ○ | ○ | ○ | ○ | ○ |

Would you recommend your doctor to your family and friends? *

- Definitely not
- Probably not
- Not sure
- Probably yes
- Definitely yes

## Thinking about the future

**Thank you for telling us about your experiences. In the following questions, please think about the future…**

If you are given a choice of doctors or health plans in the future, how important would open notes be in your decision? *

- Not important
- Somewhat important
- Very important

---

In the future, I should be able to add my own comments to the doctor's note *

- Disagree
- Somewhat disagree
- Somewhat agree
- Agree
- No opinion

---

In the future, I should be able to approve what's written in the note. *

- Disagree
- Somewhat disagree
- Somewhat agree
- Agree
- No opinion

---

# Thinking about the future

In the future, notes from hospital admissions should be available on PatientSite. (Being admitted usually means that you stay in the hospital at least one night.) *

- Disagree
- Somewhat disagree
- Somewhat agree

- Agree
- No opinion

---

In the future, I would like the option of letting family members or friends who help me with my health care have their own access to my visit notes. *

- Disagree
- Somewhat disagree
- Somewhat agree
- Agree
- No opinion

---

Do you have any other changes or suggestions about open notes?

---

## About you

In general, how would you rate your overall health?
*

- Excellent
- Very Good
- Good
- Fair
- Poor

---

What is the highest grade or level of school that you have completed? *

- 8th grade or less
- Some high school, but did not graduate

- High school graduate or GED
- Some college or 2-year degree
- 4-year college graduate
- Some post-baccalaureate graduate school
- Masters or Doctoral degree

## About you

Are you of Spanish/Hispanic/Latino ethnicity?

- Yes
- No

What do you consider to be your racial background? *(Check all that apply)*

- White
- Black or African-American
- American Indian or Alaskan Native
- Asian
- Native Hawaiian or Pacific Islander
- Other

Which of the following best describes your current employment status? *(Check all that apply)*

- Employed for wages
- Self-employed
- Homemaker
- Unemployed
- Retired
- Unable to work
- Prefer not to answer

If there is anything else that you would like us to know about you or other comments you would like to make, please write them here.

## Thank You!

Thank you for completing the OpenNotes survey! Please close your browser to exit.
