# Supplementary material for: Online Access to Doctors' Notes: Patient Concerns About Privacy
Source: J Med Internet Res. 2013 Sep 26;15(9):e208. doi: 10.2196/jmir.2670 (PMC3785972; doi:10.2196/jmir.2670)
Supplement: Supplementary file 3 [file jmir_v15i9e208_app3.pdf]

Multimedia Appendix 3. Individual patient matched baseline and post-intervention response to survey question[a]: "I am concerned about my privacy" (N=3,874)

		Post-Intervention					
		Disagree	Somewhat disagree	Somewhat agree	Agree	Don't know	Total[b]
Baseline (Before Intervention)	Disagree	1230 (66.3%)	174 (9.4%)	192 (10.4%)	213 (11.5%)	44 (2.4%)	1853 (100%)
	Somewhat disagree	213 (37.7%)	140 (24.8%)	116 (20.5%)	81 (14.3%)	15 (2.7%)	565 (100%)
	Somewhat agree	271 (31.4%)	129 (15.0%)	283 (32.8%)	155 (18.0%)	24 (2.8%)	862 (100%)
	Agree	70 (16.9%)	23 (5.6%)	113 (27.4%)	199 (48.2%)	8 (1.9%)	413 (100%)
	Don't know	75 (41.4%)	26 (14.4%)	39 (21.5%)	28 (15.5%)	13 (7.2%)	181 (100%)
	Total	1859	492	743	676	104	3874

[a]Baseline question: "If I could read my doctor's notes, I would be concerned about my privacy."

Post-intervention question: "As a result of reading/having access to my doctor's notes, I am concerned about my privacy."

[b]Row percents total to 100%
